# Prognostic Value of Molecular Markers and Implication for Molecular Targeted Therapies in Nasopharyngeal Carcinoma: An Update in an Era of New Targeted Molecules Development

**DOI:** 10.14740/wjon610w

**Published:** 2015-02-14

**Authors:** Mu-Tai Liu, Mu-Kuan Chen, Chia-Chun Huang, Chao-Yuan Huang

**Affiliations:** a Department of Radiation Oncology, Changhua Christian Hospital, 135 Nan Shiau Street, Changhua, Taiwan 500, ROC; b Department of Oncology, National Taiwan University Hospital, 7 Chung San South Road, Taipei, Taiwan 100, ROC; c Department of Medicine, Chang Shan Medical University, 110 Section 1, Chien- Kuo N. Road, Taichung, Taiwan 402, ROC; d Department of Radiology, Yuanpei University of Science and Technology, 306 Yuanpei Street, Hsinchu, Taiwan 300, ROC; e Department of Otorhinolaryngology, Head and Neck Surgery, Changhua Christian Hospital, 135 Nan Shiau Street, Changhua, Taiwan 500, ROC

**Keywords:** Nasopharyngeal carcinoma, Molecular marker, Targeted therapy

## Abstract

The aim of the study was to evaluate the prognostic significance of molecular biomarkers which could provide information for more accurate prognostication and development of novel therapeutic strategies for nasopharyngeal carcinoma (NPC). NPC is a unique malignant epithelial carcinoma of head and neck region, with an intimate association with the Epstein-Barr virus (EBV). Currently, the prediction of NPC prognosis is mainly based on the clinical TNM staging; however, NPC patients with the same clinical stage often present different clinical outcomes, suggesting that the TNM stage is insufficient to precisely predict the prognosis of this disease. In this review, we give an overview of the prognostic value of molecular markers in NPC and discuss potential strategies of targeted therapies for treatment of NPC. Molecular biomarkers, which play roles in abnormal proliferation signaling pathways (such as Wnt/β-catenin pathway), intracellular mitogenic signal aberration (such as hypoxia-inducible factor (HIF)-1α), receptor-mediated aberrations (such as vascular endothelial growth factor (VEGF)), tumor suppressors (such as p16 and p27 activity), cell cycle aberrations (such as cyclin D1 and cyclin E), cell adhesion aberrations (such as E-cadherin), apoptosis dysregualtion (such as survivin) and centromere aberration (centromere protein H), are prognostic markers for NPC. Plasma EBV DNA concentrations and EBV-encoded latent membrane proteins are also prognostic markers for NPC. Implication of molecular targeted therapies in NPC was discussed. Such therapies could have potential in combination with different cytotoxic agents to combat and eradicate tumor cells. In order to further improve overall survival for patients with loco-regionally advanced NPC, the development of innovative strategies, including prognostic molecular markers and molecular targeted agents is needed.

## Introduction

**Figure 1:**
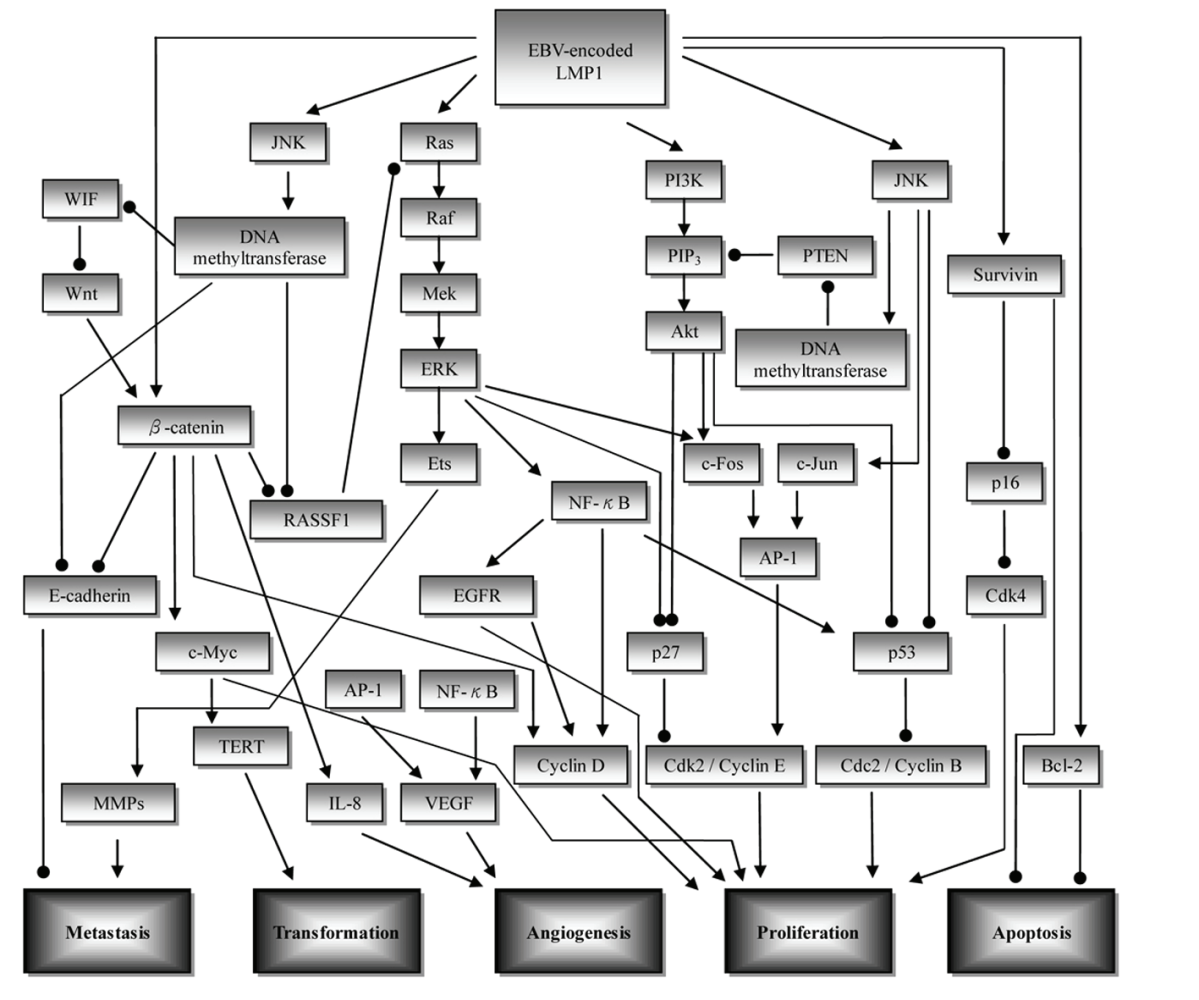
Overview of the signaling pathways involved in nasopharyngeal carcinoma (NPC) development. Initiation of upstream signaling proteins in the NPC development begins with LMP1. Subsequent induced activity of downstream proteins in several pathways such as β-catenin, NF-κB, and AP-1 leads to dysregulation of cell proliferation (CDK/cyclin protein), increase in angiogenesis (VEGF, IL-8), metastasis (E-cadherin, MMPs), cell transformation (TERT), and inhibition of apoptosis (survivin, Bcl-2). ——►: stimulatory effect; ——●: inhibitory effect; AP-1: activator protein 1; EGFR: epidermal growth factor receptor; ERK: extracellular signal related kinase; JNK: c-Jun N-terminal kinase; LMP1: latent membrane protein 1; MMP: matrix metalloproteinase; PTEN: phosphatase and tensin homolog; PI3K: phosphoinositol-3-kinase; RASSF: Ras association domain family; TERT: telomerase reverse transcriptase; VEGF: vascular endothelial growth factor; WIF: Wnt inhibitory factor.

Nasopharyngeal carcinoma (NPC) is a unique malignant epithelial carcinoma of head and neck region, with an intimate association with the Epstein-Barr virus (EBV) [1-4]. According to International Agency for Research on Cancer, there were 84,400 cases of NPC, and 51,600 deaths from it, in 2008. The worldwide distribution of NPC is extremely unbalanced, with an age-standardized incidence rate of 20 - 50 per 100,000 males in southern China to 0.5 per 100,000 in mainly white populations [5]. Currently, the prediction of NPC prognosis is mainly based on the clinical TNM staging, however, NPC patients with the same clinical stage often present different clinical outcomes, suggesting that the TNM stage is insufficient to precisely predict the prognosis of this disease [6]. Several studies showed that the biological behavior and prognosis could be different in the NPC patients with the same classification [7, 8]. One elemental factor mediating the biological behavior of NPC is the alteration of molecular signaling pathways. Such signaling pathways of NPC are critical for cell survival, growth, and metastasis. Therefore, it is important to search for novel molecular biomarkers, which could provide more accurate prognostication and develop therapeutic intervention for NPC patients. The schematic representation of the signaling pathways is shown in Figure 1. A table depicting the pathways and molecular targeted therapy involved is shown in Table 1. Ranking of the potential importance of the pathways is described as follows: rank 1: well-designed cohort analytic studies; rank 2: comparative or correlation studies; rank 3: evidence obtained from research in a small number of studies.

**Table 1:** Molecular Pathways and Targeted Therapeutic Agents Involved in Nasopharyngeal Cancer

Mechanism	Pathway	Targeted therapeutic agent or strategy	Clinical application	In development
Abnormal signaling pathways	EGFR	Cetuximab: anti-EGFR antibody	+	
	PI3K/AKT and PTEN	PI3K inhibitor: Y294002		+
	RKIP	RKIP: alter radiosensitivity		+
	RASSF	Demethylation of RASSF2A		+
	Wnt/β-catenin	YC-1: anti-invasion activity		+
Receptor-mediated aberration	VEGF	Bevacizumab: monoclonal antibody against VEGF	+	
	c-MET	c-MET inhibitor: SU11274, BAY 853474, PF-04217903		+
Intra-cellular mitogenic signals aberration	HIF-1α	HIF-1α inhibitor: NSC-134754, pantoprazole		+
Cell cycle aberration	Cyclin-dependent kinase (CDK), cyclin D1, E	Cyclin-dependent kinase inhibitor: seliciclib	+	
	c-Myc	c-Myc inhibitor		+
	MicroRNAs	miRNAs of let-7 family: suppress cell proliferation		+
Cell adhesion aberration	E-cadherin	DNA demethylating agent: 5-aza-dC		+
	Matrix metalloproteinases (MMPs)	Synthetic inhibitors: marimastat, tanomastat (BAY12-9666), prinomastat (AG3340)		+
		Recombinant human endostatin: endosatr		+
		Off-target inhibitors: bisphosphonates	+	
		Natural inhibitors: neovastat (AE-941)		+

## Rank 1

### Abnormal proliferation signaling pathways

#### Epidermal growth factor receptor (EGFR)

EGFR is a 170 kD transmembrane glycoprotein receptor with an intrinsic tyrosine kinase activity that regulates cell growth. Growth factor receptors with tyrosine kinase activity regulate fundamental cell behavior including cell survival, differentiation, motility, and proliferation. The type I growth factor receptor family consists of EGFR, HER-2/neu (c-erbB2), and HER-3/neu (c-erbB3). These receptors consist of a cysteine-rich extracellular domain, a single transmembrane spanning region, and a cytoplasmic tail containing tyrosine kinase activity and several tyrosine residues that are phosphorylated on ligand binding. The resulting signal transduction through multiple intracellular pathways then leads to cell proliferation and other key events that may affect tumor progression [9]. EGFR overexpression has been found in over 80% NPC cases [9, 10], and was associated with tumor metastasis, recurrence, and poor survival in patients with NPC [10, 11]. EGFR extent > 25% was associated with a significantly poorer treatment outcome. The 5-year disease-specific survival, relapse-free survival, loco-regional relapse-free, and distant metastasis-free rates in patients with EGFR extent > 25% were 48%, 36%, 60%, and 55%, respectively. The corresponding rates in patients with EGFR extent < 25% were 86%, 80%, 93%, and 86%. The differences were all statistically significant, except for distant metastasis. In multivariate analysis, EGFR extent was the only independent factor that predicted for disease relapse, loco-regional failure, and death resulting from cancer. The possible efficacy of molecular targeted therapy against EGFR, especially in advanced stage disease, should also be examined [7]. Cao et al evaluated the prognostic value of expression of EGFR and nm23 in 127 patients with advanced stage NPC. The positive EGFR expression had a higher recurrent rate than the negative (P = 0.015). Negative expression of EGFR had a significantly better 5-year overall survival (OS) and disease-free survival (DFS) than positive expression (P = 0.015, 0.013, respectively) [12]. These data suggested that EGFR and nm23 can serve as reliable biomarkers for prognosis prediction in patients with NPC who may benefit from alternate treatment strategy and targeted treatment. Yang and colleagues investigated the difference of expression of EGFR and Ki67 in primary and recurrence of NPC to supply a micro-evidence of anti-EGFR targeted maintenance therapy for NPC. There was a significantly shorter time to loco-regional relapse in patients with positive expression of EGFR than patients with negative EGFR expression in primary (P = 0.010) and in relapse (P = 0.022). A strongly significant correlation between EGFR and Ki67 molecules expression was obtained in primary (r = 0.573; P = 0.001) and in recurrent focus (r = 0.698; P = 0.000). These findings suggest that the recurrent NPC cells that survive after radiotherapy express a chemoradiation-induced cytoprotective phenotype that is overexpression of EGFR and Ki67. These findings have clinical significance and provide further support for carrying out clinical trials to apply the use of maintenance cetuximab after chemoradiotherapy to increase curability and prolong the disease-free interval in patients with tumors that are dependent on EGFR [13].

##### 1) Clinical application of molecular targeted therapy: cetuximab (anti-EGFR antibody) and gefitinib (EGFR tyrosine kinase inhibitor)

Cetuximab is an anti-EGFR antibody that has been shown to improve survival when combined with RT in patients with loco-regionally advanced non-NPC head and neck squamous cell carcinoma (HNSCC) [14]. Chan and colleagues reported that cetuximab in combination with carboplatin demonstrated clinical activity and an acceptable safety profile in heavily pretreated patients with recurrent or metastatic NPC who had previously experienced treatment failure with platinum-based therapy. The overall response rate (ORR) was 12% and the stable disease (SD) rate was 48% [15-21] (Table 2). Two phase II trials utilizing the EGFR tyrosine kinase inhibitor gefitinib as a single agent have reported no evidence of objective response, while SD rates ranged from 11% to 19% [17, 18,] (Table 2). Ma and colleagues evaluated the feasibility of adding cetuximab to concurrent cisplatin and intensity-modulated radiotherapy (IMRT) in loco-regionally advanced NPC. Thirty patients with American Joint Committee on Cancer stage III-IVB NPC were given an initial dose of cetuximab (400 mg/m^2^) 7 - 10 days before receiving concurrent IMRT, weekly cisplatin (30 mg/m^2^/week) and cetuximab (250 mg/m^2^/week). Grade 3-4 oropharyngeal mucositis occurred in 26 (87%) patients and 10 (33%) patients required short-term nasogastric feeding. Three patients (10%) had grade 3 cetuximab-related acneiform rash. At a median follow-up of 31.8 months, the 2-year progression-free survival (PFS) was 86.5% (95% confidence interval (CI): 74.3 - 98.8). Concurrent administration of cetuximab, weekly cisplatin and IMRT is a feasible strategy against loco-regionally advanced NPC. Preliminary survival data compared favorably with historic data [19] (Table 1, 2).

**Table 2:** Clinical Trials of Molecular Targeted Therapy in Nasopharyngeal Cancer

Agent	Phase	Treatment	Number of patients	ORR (%)	CR (%)	SD (%)	TTP (mo)	PFS (mo)	OS (mo)	Reference
Single or combination therapy in metastatic/recurrent disease										
Cetuximab	II	Cetuximab 250 mg/m^2^ weekly (400 mg/m^2^ loading dose) + carboplatin	60	11.7	0	48.3	2.7	-	7.8	Chan et al, 2005 [16]
Gefitinib	II	Gefitinib 250 mg daily	19	0	0	10.5	4	-	16	Chua et al, 2008 [17]
Gefitinib	II	Gefitinib 500 mg daily	16 (15 evaluable)	0	0	18.8	2.7	-	12	Ma et al, 2008 [18]
Single or concurrent therapy in locally advanced disease										
Seliciclib	I	800 mg or 400 mg twice daily on days 1 to 3 and 8 to 12	20 (14 evaluable)	0	0	Seven patients had > 25% reduction of tumor size	-	-	-	Hsieh et al, 2009 [19]
Cetuximab	II	An initial dose of cetuximab (400 mg/m^2^) 7 - 10 days before receiving concurrent IMRT, weekly cisplatin (30 mg/m^2^/week) and cetuximab (250 mg/m^2^/week)	30	96	90	-	-	2-year PFS 86.5%	2-year OS 89.9%	Ma et al, 2012 [20]
Bevacizumab	II	Three cycles of bevacizumab (15 mg/kg) and cisplatin (100 mg/m^2^) both given on days 1, 22, and 43 of IMRT, then received three cycles of bevacizumab (15 mg/kg) and cisplatin (80 mg/m^2^), both given on days 64, 85, and 106 after IMRT, and three cycles of fluorouracil (1,000 mg/m^2^/day), given on days 64 - 67, 85 - 88, and 106 - 109 after IMRT	46 (44 evaluable)	91	-	-	-	2-year PFS 74.7%	2-year OS 90.9%	Lee et al, 2012 [21]

ORR: overall response rate; CR: complete response; SD: stable disease; SD: stable disease; TTP (mo): time to progression (month); PFS (mo): progression-free survival (month); OS (mo): overall survival (month).

### Receptor-mediated aberrations

#### Vascular endothelial growth factor (VEGF)

VEGF is a member of the platelet-derived growth factor/VEGF family that specifically acts on endothelial cells. It promotes the proliferation of vascular endothelial cells and angiogenesis; in the meantime, the permeability of the vessels is increased and the leakage of intravascular contents is facilitated, thereby providing matrix for the migration of endothelial cells and vascular formation, and these mechanisms are paramount for tumor development and metastasis [22]. The association of VEGF protein expression in tumor tissues with lymph node metastasis, disease recurrence, advanced TNM classification, and poorer prognosis of patients with NPC has been investigated in several studies. Wakisaka et al reported that VEGF expression in tumor tissue is positively correlated with cervical lymph node metastasis and microvascular density [23]. Krishna et al reported that overexpression of VEGF was seen in 67% of NPC cases. Higher expression of VEGF in NPC patients was related to higher rate of recurrence, nodal positivity and lower survival [24]. Pan et al reported that the TNM classification of NPC is associated with VEGF expression levels in the tumor tissues [11]. Li et al reported that expression of survivin and VEGF were significantly associated with TNM stage, T-stage and metastasis of NPC. The patients with survivin and VEGF overexpression presented lower 5-year survival rate, as compared to those of low-expression (42.32% vs. 70.54%, 40.1% vs. 67.8%, respectively, P < 0.05), especially in advanced stage patients (36.51% vs. 73.41%, 35.03% vs. 65.22%, respectively, P < 0.05) [6].

Chang and colleagues found that the levels of VEGF, interleukin (IL)-6, IL-8, interferon-inducible protein 10 (IP-10), tumor necrosis factor (TNF)-α, and macrophage inflammatory protein (MIP)-3α were significantly elevated in patients with NPC; the patients with NPC with higher levels of VEGF, IL-8, MIP-3α, and EBV DNA had worse prognoses for OS (P = 0.035, 0.008, 0.005, and 0.007, respectively) [25]. Lv and colleagues found that the serum VEGF (sVEGF) was positively associated with histology, TNM classification, distant metastasis, and OS (P < 0.05). The 4-year OS and distant metastasis-free survival (DMFS) of the high-sVEGF versus low-sVEGF groups were 68% versus 86% and 70% versus 89%, respectively (P < 0.05). Stratified analysis showed that patients with stage IV(a,b), T(4), N(1), or N(positive) disease with high VEGF levels had worse 4-year OS and 4-year DMFS than those with low VEGF levels (P < 0.05). Multifactorial Cox regression confirmed sVEGF was among the independent prognostic factors [22].

##### 1) Clinical application of molecular targeted therapy: bevacizumab (a monoclonal antibody directed against VEGF)

Because the predominant pattern of failure in loco-regionally advanced NPC is distant metastasis and because patients with increased VEGF have a higher likelihood of recurrence, distant metastases, and decreased survival, Lee and coworkers did a phase 2 multi-institutional trial (RTOG 0615) to test the addition of bevacizumab (a monoclonal antibody directed against VEGF) to standard chemoradiation treatment for this group of patients. No grade 3-4 hemorrhages or grade 5 adverse events were recorded. With a median follow-up of 2.5 years, the estimated 2-year loco-regional progression-free interval was 83.7% (95% CI: 72.6 - 94.9), the 2-year distant metastasis-free interval was 90.8% (82.2 - 99.5), the 2-year PFS was 74.7% (61.8 - 87.6), and 2-year OS was 90.9% (82.3 - 99.4). The addition of bevacizumab to standard chemoradiation treatment for patients with NPC is feasible, and might delay the progression of subclinical distant disease [21] (Table 1, 2).

### Plasma EBV DNA concentrations

Lo et al investigated the prognostic implication of pretreatment plasma/serum EBV DNA concentration in NPC. Those with recurrence or metastasis within the first year after treatment had a higher median plasma EBV DNA concentration than those without events (41,756 copies/mL versus 5,807 copies/mL; P < 0.001, Mann-Whitney rank-sum test). Plasma EBV DNA was an independent prognostic indicator for early clinical events (relative risk = 3.8 (95% CI: 1.6 - 9.2 for each 10-fold increase in plasma EBV DNA concentration; P = 0.003)). Circulating EBV DNA was found to be a significant variable associated with NPC-related death in multivariate Cox’s regression analysis (relative risk = 1.6 (95% CI: 1.1 - 2.1 for each 10-fold increase in serum EBV DNA concentration; P = 0.007)) [26]. Leung et al demonstrated that pretherapy circulating EBV DNA load was an independent prognostic factor to International Union Against Cancer (UICC) staging in NPC patients [27]. Lin et al evaluated the prognostic value of plasma EBV DNA concentrations in patients with advanced NPC. The median concentrations of plasma EBV DNA were 681 copies/mL among 25 patients with stage III disease, 1,703 copies/mL among 74 patients with stage IV disease, and 291,940 copies/mL among 19 control patients with distant metastasis (P < 0.001). Patients with relapse had a significantly higher plasma EBV DNA concentration before treatment than those who did not have a relapse (median, 3,035 vs. 1,202 copies/mL; P = 0.02). The plasma EBV DNA concentration was persistently low or undetectable in patients with a complete clinical remission. OS (P < 0.001) and relapse-free survival (P = 0.02) were significantly lower among patients with pretreatment plasma EBV DNA concentrations of at least 1,500 copies/mL than among those with concentrations of less than 1,500 copies/mL. Patients with persistently detectable plasma EBV DNA had significantly worse OS (P < 0.001) and relapse-free survival (P < 0.001) than patients with undetectable EBV DNA 1 week after the completion of radiotherapy [28].

Chai et al investigated the clinical significance of plasma EBV DNA loads in a large and multi-ethnic cohort of Malaysian patients with NPC. The median level of plasma EBV DNA in stage IV patients with distant metastasis was > 9-fold higher than those without systemic spread (P = 0.001), suggesting plasma EBV DNA measurement could aid in the diagnosis of metastatic disease in advanced cases. Using a cut-off value of 8,000 copies/mL, the authors demonstrated that EBV DNA level was a strong predictor for OS of NPC patients [2]. Ferrari et al evaluated the role of plasma EBV DNA levels in predicting recurrence of NPC in a cohort of Western patients with stage IIb-IVb nasopharyngeal cancer. Pre-treatment levels significantly correlated with the initial stage and probability of relapse. Their increase was 100% specific and 71.3% sensitive in detecting loco-regional or metastatic recurrence (an overall accuracy of 94.4%). The results of this study confirm that patients from a Western country affected by loco-regionally advanced NPC have high plasma EBV DNA levels at diagnosis. The monitoring of plasma levels is sensitive and highly specific in detecting disease recurrence and metastases [29]. Li et al reported that plasma EBV DNA concentrations and Raf kinase inhibitory protein (RKIP) were independent prognostic markers for 5-year DMFS [30].

## Rank 2

### Abnormal proliferation signaling pathways

#### Wingless-type (Wnt)/β-catenin pathway

Aberrant activation of Wnt signaling pathway plays a critical role in oncogenesis of various human cancers [31]. Epigenetic inactivation of negative Wnt/β-catenin signaling regulators leads to the aberrant activation of this signaling pathway in NPC tumorigenesis. Wnt inhibitory factor 1 (WIF-1), a secreted antagonist of the Wnt pathway, is frequently methylated in primary NPC tumors. With treatment of DNA demethylation reagent, WIF-1 expression is restored, highlighting a direct role of epigenetic inactivation. Ectopic expression of WIF-1 in NPC cells resulted in significant inhibition of tumor cell colony formation efficiency. Therefore, epigenetic silencing of WIF-1 contributes to the aberrant activation of Wnt/β-catenin pathway and is involved in NPC pathogenesis [32]. PRDM (PRDI-BF1 and RIZ domain containing) proteins are zinc finger proteins involved in multiple cellular regulations by acting as epigenetic modifiers. Semi-quantitative RT-PCR showed that PRDM5 was broadly expressed in human normal tissues, but frequently silenced or downregulated in 80% (4/5) of NPC cell lines due to promoter CpG methylation. PRDM5 methylation was frequently detected by methylation-specific PCR (MSP) in 93% (43/46) nasopharyngeal tumor. PRDM5 functions as a tumor suppressor at least partially through antagonizing aberrant Wnt/β-catenin signaling and oncogene expression [33]. Fendri and colleagues examined by MSP, whether WIF-1 was inactivated in 68 NPCs, and 10 normal mucosa. The authors showed that the WIF-1 promoter was methylated in 89.7% of tumors, whereas all normal mucosa were unmethylated. The WIF-1 methylation was associated with the tumor, node, and metastasis (TNM) (P = 0.003) and the age (P = 0.014). The Wnt-5a mRNA was higher in tumors and correlated with TNM (P = 0.012). The methylation of WIF-1 contributes to the activation of the Wnt pathway in NPC [34]. Identification of more epigenetically silenced negative regulators of Wnt/β-catenin signaling pathway will facilitate the development of clinical strategies targeting NPC.

##### 1) Molecular targeted therapy in development: YC-1

YC-1 has recently been demonstrated to have potent anti-invasion and anti-metastatic activity in several cancer models. The study of Hong and colleagues revealed that invasion-related signaling proteins (β-catenin, caveolin, Src and EGFR) were found to be down-modulated by YC-1 in NPC cells [35] (Table 1).

#### Phosphatidylinositol 3-kinase (PI3K)/AKT pathways and phosphatase and tensin homolog (PTEN)

The PI3K/AKT/mammalian target of rapamycin (mTOR) network plays a key regulatory function in cell survival, proliferation, migration, metabolism, angiogenesis, and apoptosis. Phosphatidylinositol is a component of eukaryotic cell membranes. The inositol head of the phospholipid can be phosphorylated at multiple sites by phosphoinositide kinases (PIKs), which act as signal transducers involved in the regulation of multiple cell functions. The PI3K superfamily has been much studied since the discovery of PI3K activity associated with viral oncoproteins and its role in growth regulation and prevention of apoptosis and other cellular responses. AKT is a key regulator of a variety of proteins involved in cell proliferation, metabolism, survival, invasion, migration, apoptosis, and DNA repair. AKT and its isoforms AKT-1, AKT-2, and AKT-3 have cell-transforming properties through the phosphorylation of multiple protein targets including mTOR, Bad, caspase 9, tuberin, GSK3b, and forkhead transcription factors involved in cell survival and apoptosis [36]. Hui et al have identified high frequency of copy number gain or amplification on chromosomes 3q (70%) in NPC. The PIK3CA gene at 3q26.32 was found to be one of the candidate oncogenes in NPC. Amplification and overexpression of PIK3CA were frequently detected in NPC [37]. Fendri et al reported that PIK3CA gene amplification was found in 21.6% of cases and was strongly associated with distant metastasis (P = 0.002), lymph node involvement (P = 0.032), and advanced tumor stage (P < 0.001) [38]. LY294002 is chemical inhibitor of PI3K, which has been used extensively to study the role of PI3K/AKT pathway in normal and transformed cells. Inactivation of PI3K using LY294002 has been demonstrated to lead to the dephosphorylation of AKT at both T308 and S473, consequently inducing specific G1 arrest in cell growth and finally to cell apoptosis [39]. Jiang and colleagues reported that LY294002 inhibited the phosphorylation of AKT (S473), cell proliferation, and induced apoptosis in human NPC cell line CNE-2Z cells. PI3K inhibitor, LY294002, might be a potentially useful therapeutic agent for NPC patients [39].

##### 1) Molecular targeted therapy in development: PI3K inhibitor, LY294002

PI3K inhibitor, LY294002, inhibited the phosphorylation of AKT (S473), cell proliferation, and induced apoptosis in human NPC cell line CNE-2Z cells. LY294002 might be a potential targeted therapeutic agent for NPC patients (Table 1).

### Latent membrane proteins (latent membrane protein 1 (LMP1) and LMP2A) of EBV

EBV-encoded LMP1 has been known to have oncogenic properties during latent infection in NPC [40]. It is required for cell immortalization and is present in 80-90% of NPC tumors [41]. The LMP1 molecule includes six transmembrane domains and a carboxy-terminus containing two signaling domains called C-terminal activating regions 1 and 2 (CTAR 1 and 2). The transmembrane domains allow LMP1 to associate with the host membrane, whereas the CTAR regions directly activate a number of signaling pathways including nuclear factor κ-B (NF-κB), mitogen-activated protein (MAP) kinases, and PI3K [42]. LMP1-positive cells have greater mobility, leading to higher metastatic potential and faster disease progression [43, 44].

Chew and colleagues suggested that EBV LMP1 was able to confer resistance of apoptosis and increased matrix metalloproteinase (MMP)-9 production in NPC cells [45]. LMP2A downregulates the NF-κB transcription factor and can decrease LMP1 expression [46]. Acting as a signal regulator, LMP2A can enhance invasiveness and motility of NPC cells through ERK/Fra-1-mediated induction of MMP-9 [47]. Chen and colleagues reported that the 5-year OS rate in patients with high LMP1 expression (n = 141) was 54%, and with low LMP1 expression (n = 83) was 68% (P = 0.020); the 5-year OS rate for NPC patients with high p-P70S6K expression (n = 106) was 49%, and low p-P70S6K expression (n = 118) was 69% (P = 0.049); the 5-year OS rate in patients with high p-4EBP1 expression (n = 128) was 49%, and low p-4EBP1 expression (n = 95) was 71% (P = 0.010). Expression of LMP1 and the genes in the mTOR pathway (p-P70S6K, p-4EBP1) significantly correlated with OS of NPC patients. However, only LMP1 was an independent prognostic factor [48]. Zhao and colleagues reported the cumulative metastasis rates were 66.75% (269/403) in cases with LMP1 expression and 46.98% (148/315) in those without LMP1 expression. LMP1 expression is positively associated with metastasis in NPC [49].

### Intracellular mitogenic signal aberration

#### HIF-1α

HIF-1 is a heterodimeric basic helix-loop-helix transcription factor composed of two subunits, HIF-1α and HIF-1β [50]. In normoxia, HIF-1α protein is degraded within minutes, but under hypoxic conditions, this protein is stabilized and upregulated [51]. Tumor hypoxia is associated with resistance to radiotherapy and chemotherapy, and HIF-1α is a key hypoxia-inducible transcriptional factor that upon activation, leads to the upregulation of several important hypoxia-responsive genes that regulate apoptosis, glucose metabolism (e.g. carbonic anhydrase (CA)-9), and angiogenesis (e.g. VEGF receptor (VEGFR) and its ligands) [52]. Hui et al showed that HIF-1α and VEGF overexpression occurred in around 60% of 90 patients with advanced NPC, and patients whose tumors displayed a high level of HIF-1α expression or HIF-1α-VEGF co-expression had a worse clinical outcome than patients whose tumors did not [53]. Chan et al also found that HIF-1α-VEGF co-expression was correlated with poorer PFS in patients with advanced NPC [54]. Xueguan et al reported that the expression of HIF-1α and EGFR are associated with a poor survival and increased occurrence of tumor invasion and distant metastases in patients with NPC receiving radiotherapy [55]. Recent studies have suggested that autophagy plays a pivotal role in regulation of cancer development and progression. High expression of the autophagy-related Beclin 1 protein predicted favorable patient outcome in several tumors. Wan et al found that higher Beclin 1 expression predicted poorer OS, PFS and DMFS. Moreover, a positive relationship between HI F-1α and Beclin 1 expression was found. Among patients with elevated HI F-1α expression, a subset with lower Beclin 1 expression displayed a significant OS advantage than those with higher expression (P = 0.036). HIF-1α-associated Beclin 1 high expression might facilitate NPC cells surviving from chemoradiotherapy, suggesting a novel therapeutic molecular target for NPC [56]. The HIF-1α and Forkhead Box O3a transcription factor (FOXO3a) have been reported to play important roles in the development and prognosis of human cancers. Shou et al found that FOXO3a was low expressed and HIF-1α was high expressed in NPC tissues, compared with normal nasopharyngeal tissues (both P < 0.05). The low expression of FOXO3a was significantly correlated with clinical stage (P = 0.003), T stage (P = 0.011), lymph node metastasis (P = 0.003), and distant metastasis (P = 0.030), and overexpression of HIF-1α was significantly correlated with T stage (P = 0.026), lymph node metastasis (P = 0.002), and distant metastasis (P = 0.010). The Spearman analysis indicated that FOXO3a expression was inversely correlated with HIF-1α expression (P < 0.001). OS curves estimated by Kaplan-Meier showed that tumor patients with low FOXO3a or high HIF-1α expression had significantly poorer prognosis compared with patients with high FOXO3a or low HIF-1α levels (P < 0.001, and P = 0.012, respectively) [57].

##### 1) Molecular targeted therapy in development: HIF-1α inhibitor: NSC-134754, pantoprazole

Baker and colleagues investigated the effects of the small-molecule HIF-pathway inhibitor NSC-134754. NSC-134754-treated tumors revealed lower expression of HIF-1α. NSC-134754 induced metabolic alterations *in vitro* and early anti-tumor activity *in vivo* [58] (Table 1). *In vitro* and *in vivo* our study revealed that pantoprazole (PPZ) inhibited tumor cells proliferation, induced apoptosis and decreased the expression of HIF-1α protein. PPZ could suppress tumor growth acting as an HIF-1α protein inhibitor [59] (Table 1).

### Receptor-mediated aberration

#### Mesenchymal-epithelial transition factor (c-MET)

c-MET is a membrane-associated tyrosine kinase that is located upstream of several important oncogenic pathways [52]. MET tyrosine kinase is important in various cellular functions including proliferation, mitogenesis, formation of branching tubules, angiogenesis, and tumor cell invasion and metastasis [60]. LMP1 could cause overexpression of c-MET by induction of transcription factor Ets1 [61]. There is also *in vitro* evidence suggesting cross-talk between the c-MET and EGFR pathways wherein EGFR activation can phosphorylate and activate c-MET [62]. The activation of the receptor tyrosine kinase c-MET in cancer correlates with poor prognosis, where aberrantly active c-MET triggers tumor growth, angiogenesis and metastasis [63]. There are several c-MET inhibitors in development, e.g. SU11274, BAY 853474, and PF-04217903 [64-66] (Table 1).

In NPC patients, c-MET protein expression is present in 52-72% of patients, associated with cervical nodal metastases and poor prognosis [67, 68]. Qian et al reported that high MET protein expression correlated with poorer survival in late-stage NPC and served as an independent prognostic indicator. In their study, the mean survival time was 118 months in the low MET expression group versus 52 months in the high expression group (P < 0.01). The study of Kim et al showed that high MET expression was a statistically significantly negative prognostic factor on OS of patients with NPC. Patients with high (> 50%) MET expression showed worse 5-year OS rate than that of patients with low MET expression (48% vs. 84%, P = 0.02, HR = 5.56, 95% CI: 1.18 - 26.06) [60].

##### 1) Molecular targeted therapy in development: c-MET inhibitors

There are several c-MET inhibitors in development, e.g. SU11274, BAY 853474, and PF-04217903 (Table 1).

### Tumor suppressors

#### p16 activity

p16 is a cyclin-dependent kinase inhibitor, also known as CDKN2A, a tumor suppressor protein, which in humans is encoded by the CDKN2A gene [69, 70]. p16 is frequently inactivated in many human cancers [71, 72]. NPC cell lines have low levels of p16 secondary to hypermethylation of the p16 [73]. This epigenetic alteration may be mediated by LMP1-induced formation of a c-Jun/JunB heterodimer causing the activation of DNA methyl-transferase [74]. Wang et al reported that p16 positive rate was 100% for the epithelia of chronic inflammation of nasopharynx. It was significantly higher than the p16 positive rate for the carcinoma of nasopharynx (38.4%, P < 0.01). There was significant difference of p16 positive expression in differentiation of NPC (poor differentiation versus undifferentiation), clinical staging (I-II versus IV) and grading of tumor (T1-T2 versus T3, T4) (P < 0.01). The 3-year survival rates were 88.9% and 72.9% in p16 expression (+) and (-) patients respectively (P < 0.05) [75]. Makitie et al found when p16 expression was analyzed controlling for age, weight loss, and stage in a multivariate analysis, an association between absence of p16 expression and worse survival (P = 0.02) [76]. Xiang et al found that among the 90 NPC cases studied, 42 cases (46.7%) were negative for p16 protein. The non-expression rate of p16 protein also correlated with the 5-year survival rate. The non-expression rate was 60.0% in patients who died within 5 years, in contrast to 20.0% in those alive for over 5 years after diagnosis. The non-expression rates of p16 protein in cases with or without distant metastasis were 81.8% and 41.8% respectively (P < 0.05) [77].

#### p27 activity

p27 is a cyclin-dependent kinase inhibitor and plays an important role in negative regulation of the cell cycle during G0 and G1 phases [78]. Jab1/CSN5 promotes cell proliferation and inactivates p27 by inducing translocation of p27 from the nucleus to the cytoplasm, which accelerates p27 degradation through the ubiquitin-dependent proteasome pathway and promotes cell-cycle progression [79]. Jab1/CSN5 overexpression is correlated with the loss of p27 in several cancers and low p27 expression is associated with higher tumor grades [80]. Pan and colleagues examined the functional relationship between Jab1 and p27 protein expression in NPC. Immunohistochemical analysis showed an inverse association between Jab1 and p27 in NPC tissue samples, and overexpression of Jab1 correlated with poor survival in patients with NPC. The findings suggest that Jab1 overexpression plays an important role in the pathogenesis of NPC through Jab1-mediated p27 degradation. Jab1 therefore represents a novel diagnostic marker and therapeutic target in patients with NPC [81]. Low p27 is demonstrated in NPC as well as a number of cancers [78, 82]. Hwang and colleagues reported that in patients with NPC, 47 of 69 cases (68%) expressed low levels of p27 [81]. In the study of Pan and colleagues, 29 of 45 NPC cases (64%) expressed low or no nuclear p27, and 28 cases (62 %) expressed low or no cytoplasmic p27 [81]. A low level of p27 expression was significantly correlated with loco-regional recurrence in NPC [83].

#### nm23-H1 protein or mRNA

The nm23-H1 gene (NME1), localized on chromosome 17q21.3 was first isolated as a metastasis suppressor gene by differential screening of cDNA library from high and low metastatic clones of a murine melanoma cell line [84]. Guo et al investigated the expression levels of nm23-H1 mRNA and its protein in human NPC to clarify the relationship between nm23-H1 and metastasis and prognosis of patients with NPC. The authors found that nm23-H1-negative tumors were associated with a higher incidence of lymph node metastasis (84.2%) than nm23-H1-positive ones (32.8%, P < 0.01). The distant metastasis and loco-regional recurrence rates in the nm23-H1-negative group were 55.8% and 31.68%, respectively but only 17.2% and 11.5%, respectively, in the nm23-H1-positive group (P < 0.01). A significant association was found between expression of nm23-H1 protein and prognosis (P < 0.01). Expression of nm23-H1 protein indicated favorable prognosis, suggesting that the absence of nm23-H1 protein expression was significantly associated with lymph-node metastasis, recurrence and distant metastasis in patients with NPC [85]. Huang et al found that there were inverse correlations between the expression of p16, nm23-H1, or E-cadherin protein and lymph node metastasis (P < 0.05), indicating that the expressions of p16, nm23-H1, and E-cadherin gene were related to the prognosis of patients with NPC [86].

### Cell cycle aberrations

#### Cyclin D1

The cell cycle regulator cyclin D1 is a critical regulator of the G1/S phase transition [87]. It forms complexes with cyclin-dependent kinases (CDK) 4 or CDK6 and is responsible for the phosphorylation of the retinoblastoma tumor suppressor protein, resulting in the release of E2F transcription factors that allow cells to enter into S phase. The G1/S checkpoint is frequently altered in many epithelial tumors and may confer growth advantage and enhance tumorigenesis [88]. Nuclear EGFR could directly bind to the cyclin D1 promoter under the regulation of the oncoprotein LMP1 [89]. Lai et al reported that rates of early local recurrence (within 5 years) were significantly higher (P < 0.01) for NPC patients with high levels of cyclin D1 before radiation therapy (24 of 35 patients (68.6%)) as compared with patients with low or no expression (three of 29 (10.3%)). Furthermore, NPC patients bearing high levels of cyclin D1 had a poorer prognosis concerning 10-year survival than the others (P < 0.001). Cyclin D1 can be used as an indicator of recurrence and subsequent prognosis in NPC after radiation therapy [90].

##### 1) Clinical application of molecular targeted therapy: seliciclib

Seliciclib is a CDK inhibitor. In an early clinical trial, the use of seliciclib in 16 patients with treatment-naive locally advanced NPC resulted in SD in 14 evaluable patients. Seven of 14 evaluable patients had clinical evidence of tumor reduction. Reduced protein expressions of Mcl-1, cyclin D1 and phosphorylated retinoblastoma protein pRB were shown in some patients post-treatment, indicative of cell cycle modulation by seliciclib, more specifically inhibition of CDK2/cyclin E, CDK7/cyclin H, and CDK9/cyclin T [19] (Table 2).

#### Cyclin E

CDK are key regulators of cell cycle progression. CDK-2 and its partner, cyclin E, regulate the G1 to S cell cycle transition by phosphorylating the retinoblastoma protein. Engineered cyclin E overexpression shortens the cell cycle and causes chromosomal instability [91]. Ko et al found that high levels of cyclin E significantly correlated with late-stage NPC (P = 0.009) and a poor OS (P = 0.010). Overexpression of cyclin E messenger ribonucleic acid showed an adverse prognostic significance, correlating with an advanced stage of NPC and a low OS rate [92].

### Cell adhesion aberrations

#### E-cadherin

E-cadherin is a transmembrane glycoprotein that mediates cell communication in normal cells and is a metastasis suppressor in tumor cells [93]. The downregulation of E-cadherin in NPC is due to aberrant promoter methylation of E-cadherin gene [94, 95]. The detailed methylation profile at individual CpGs within CpG island of E-cadherin promoter region was confirmed by bisulphite sequencing [95]. E-cadherin levels are inversely proportional to disease progression; metastatic NPC tumors display lower E-cadherion mRNA and protein levels than primary NPC tumors (19% in primary tumors vs. 42% in metastatic tumors) [86]. A significant correlation was also found between E-cadherin expression and study group (P = 0.003): E-cadherin protein levels were higher in the control group than in the NPC groups. E-cadherin protein levels were also associated with metastasis (P = 0.002) and patient outcomes (P = 0.013): NPC patients with poor prognosis displayed lower levels of E-cadherin expression than those displaying neither recurrence nor metastasis [96]. E-cadherin gene could be demethylated and reactivated in HNE-1 and CNE-2 cells upon treatment with 5-aza-dC, a DNA demethylating agent. Demethylation therapy could serve as a promising strategy for NPC patients [95].

##### 1) Molecular targeted therapeutic agent in development: 5-aza-dC, a DNA demethylating agent

5-aza-dC, a DNA demethylating agent, is a potential molecular targeted therapeutic agent for NPC patients (Table 1).

#### MMPs

Degradation of extracellular matrix is crucial for malignant tumor growth, invasion, metastasis and angiogenesis. MMPs are a family of zinc-dependent neutral endopeptidases collectively capable of degrading essentially all matrix components. Elevated levels of distinct MMPs can be detected in tumor tissue or serum of patients with advanced cancer, and their role as prognostic indicators in cancer has been widely examined [97]. MMPs’ family includes more than 25 members that can be divided into collagenases (MMP-1, -8, and -13), gelatinases (MMP-2 and -9), stromelysins (MMP-3 and -10), matrilysins (MMP-7 and -26), and the membrane type MMPs (MMP-14 to -17 and -24) [98]. Among MMP families, MMP-2 (the 72-kD type IV collagenase/gelatinase A) and MMP-9 (the 92-kD type IV collagenase/gelatinase B), which selectively degrade type IV collagen, a major component of extracellular matrix, are reported to be markedly associated with the invasion and metastasis of tumor cells [99]. Horikawa and colleague found that the expression of MMP-9 had a significant positive correlation with the expression of LMP1 (P = 0.0001) and the expression of MMP-9 significantly correlated with lymph node metastasis (P = 0.0004) [99]. Wong and colleague demonstrated that a high pro-MMP-2 level was found to be significantly correlated with poorer survival and patients with plasma pro-MMP-2 below 650 ng/mL had higher 5-year survival rate of 89%, compared with 50% for patients with plasma pro-MMP-2 above 650 ng/mL [100]. Nasr and colleagues investigated the susceptibility and prognostic implications of the MMP-1 (-1607) 1G/2G and MMP-9 (-1562) C/T polymorphisms in NPCs. They demonstrated that a significantly increased risk of NPC was associated with the homozygous MMP-1 (-1607) 2G2G genotype (odds ratio (OR) = 2.27; P = 0.02). A significant association was also found between the MMP-1 (-1607) 2G2G genotype and the aggressive forms of NPC as defined by large tumor size (T3-T4), lymph node metastasis and advanced stages (III-IV) at the time of diagnosis. The MMP-1 (-1607) 2G allele showed a significant association with reduced disease-free survival for NPC patients (P = 0.03) [101]. Liu and colleague analyzed the expression of MMP-9 in NPC and its correlation with clinicopathologic features. They demonstrated that high levels of MMP-9 protein were positively correlated with the status of lymph node metastasis (N classification) (P = 0.002) and clinical stage (P < 0.001) of NPC patients. Patients with higher MMP-9 expression had a significantly shorter OS time than did patients with low MMP-9 expression [102]. He and colleagues investigated the expression levels of MMP-9 in peripheral blood mononuclear cells (PBMCs) from NPC patients and healthy controls and evaluated the potential as a prognostic biomarker for NPC patients. They reported that MMP-9 mRNA levels in PBMCs were significantly higher in NPC patients than in healthy controls (P < 0.001). Increased MMP-9 expression was associated with the following clinical characteristics: advanced clinical stage (P < 0.001), T stage (P = 0.016), N stage (P = 0.002), histology type (P = 0.037), and poor OS (P = 0.049) [103].

Categories of the potential MMP inhibitors, including synthetic inhibitors, recombinant human endostatin, off-target inhibitors and natural inhibitors, are summarized in Table 1 [104].

##### 1) Clinical application of molecular targeted therapy: MMP inhibitor, zoledronic acid

Zoledronic acid is a well-validated and highly potent nitrogen-containing bisphosphonate. Jin and colleagues reported that zoledronic acid combined with chemotherapy could reduce skeletal-related events (SREs) and improve PFS and OS for NPC patients with bone metastases [105] (Table 1).

##### 2) Molecular targeted therapy in development: MMP inhibitor, endostar

Wen and colleagues investigated the efficacy of combining radiation therapy with endostar, a recombined humanized endostatin, in human NPC and human lung adenocarcinoma xenografts. The authors reported that endostar significantly sensitized the function of radiation in antitumor and antiangiogenesis in human NPC and human lung adenocarcinoma xenografts [106] (Table 1). Peng and colleagues investigated whether the recombinant human endostatin (endostar) could enhance the inhibitory effects of radiation therapy in human NPC xenograft. The authors reported that the modulation of tumor physiology caused by endostar improved the effect of radiation treatment compared with other treatment schedules [107] (Table 1).

### Apoptosis dysregulation

#### Survivin

Baculoviral inhibitor of apoptosis repeat-containing 5 (BIRC5, also called survivin) is a member of the inhibitor of apoptosis protein (IAP) family, which plays an important role in the occurrence and progression of cancer [108]. In NPC, intranuclear survivin binds CDK4 and displaces inhibitory proteins p21 and p16, thus allowing CDK4 to initiate transcription of S phase proteins [109]. LMP1 induces survivin expression and nuclear translocation [110]. The net effect is increased S-phase transitions and cell proliferation [110]. Recently, a polymorphism in the promoter of BIRC5, -31C/G (rs9904341), was shown to influence BIRC5 expression. Ma et al observed a statistically significant increased occurrence of NPC associated with the CC genotype (OR: 1.40; 95% CI: 1.13 - 1.73; P = 0.0020) compared with the genotypes containing G allele (CG + GG genotype) [108]. Xiang and colleagues investigated the expressions and prognostic value of survivin and livin in patients with NPC. The authors demonstrated that survivin levels had prognostic significance in NPC. Patients with low survivin expression had better OS, DFS and DMFS rates than the group with high survivin expression (P = 0.0086, 0.0097, and 0.0318, respectively). Cox regression analysis confirmed that high survivin expression was related to worse prognosis in NPC patients [111]. Li and colleagues demonstrated that survivin overexpression is an independent prognostic factor for patients with NPC. The patients with survivin overexpression presented lower 5-year survival rate, as compared to those of low expression (42.32% vs. 70.54%, P < 0.05), especially in advanced stage patients (36.51% vs. 73.41%, P < 0.05). The 5-year survival rate in NPC patients with survivin and VEGF dual overexpression was significantly lower than that of patients with dual low expression (18.22% vs. 73.54%, respectively; P = 0.0003) [6].

## Rank 3

### Abnormal proliferation signaling pathways

#### AKT and fibulin-3

Fibulin-3 has the ability to suppress cell migration and invasion in NPC cancer cells by decreasing the activity of phospho-AKT. Conversely, its depletion by fibulin-3-mediated siRNAs may elevate phospho-AKT activity and significantly enhance the ability of NPC cancer cells to migrate and invade. Hwang and colleagues demonstrated that loss of fibulin-3 expression was significantly correlated with advanced tumor and lymph node-metastasis stages, and indicated a poor 5-year survival rate. Lower fibulin-3 expression is an important indicator of poor survival. It may also contribute to the development of new therapeutic strategies to block the PI3K/AKT pathway in NPC cancer cells [112].

#### PTEN

PTEN is protein tyrosine phosphatase that dephosphorylates phosphatidylinositol 3,4,5-triphosphate (PIP3), the lipid second messenger that activates AKT [113, 114]. EBV microRNAs are abundant in NPC tumors. MicroRNAs are small noncoding RNAs that can inhibit the expression of target genes through a small interfering RNA-like manner. Investigation of potential EBV microRNA target genes revealed inhibition of tumor suppressor genes, e.g. PTEN frequently involved in NPC [115]. Loss of PTEN is associated with metastatic NPC. Low PTEN is demonstrated in almost 80% of clinical stage III-IV tumors versus about 20% of clinical stage I-II tumors [116].

#### Polycomb group (PcG)

Polycomb group (PcG) proteins are epigenetic gene-silencing proteins that have been implicated in embryonic development and oncogenesis. Numerous studies have shown that PcG proteins are frequently dysregulated in various cancer types and strongly correlate with an invasive or metastatic phenotype. PcG protein B lymphoma Mo-MLV insertion region 1 homolog (Bmi-1) is the first functionally identified PcG member. PTEN is a direct target of Bmi-1. Bmi-1 binds to the PTEN locus and downregulates PTEN expression, which consequently activates the PI3K/AKT pathway, stabilizes Snail, and downregulates E-cadherin, ultimately leading to enhanced invasiveness of epithelial cells [117]. Song et al demonstrated that the 5-year survival rate was 84.2% in the Bmi-1-negative group, whereas it was only 47.6% in the Bmi-1-positive group. There was a significantly higher 5-year survival rate in the Bmi-1-negative group than in the Bmi-1-positive group by log-rank test (P = 0.019). Upregulation of Bmi-1 correlates with invasion of NPCs and poor prognosis in patients [118].

#### Extracellular signal-regulated kinase (ERK)

The principal EBV oncoprotein, LMP1, has been suggested to contribute to the highly invasive nature of NPC. Signal transducer and activator of transcription 3 (STAT3) is a master transcriptional regulator in proliferation and apoptosis and is newly implicated in angiogenesis and invasiveness, which, in turn, are likely to contribute to the highly invasive character of NPC. Wang et al showed that LMP1 signals the Janus kinase 3 (JAK3) and extracellular signal-regulated kinase 1/2 (ERK1/2) pathways upon the activation of STAT3 as well as STAT transactivation activity. LMP1 induces VEGF expression via the JAK/STAT and mitogen-activated protein kinase (MAPK)/ERK signaling pathways. LMP1 can directly activate Ras, thus initiating the signaling cascade resulting in ERK activation [119]. Wang and colleagues reported that there was a statistically significant association between positive p-ERK expression and advanced clinical stage. Positive p-ERK expression was correlated with poorer OS, DFS and time to progression (TTP) [120].

#### RKIP

RKIP is a metastasis suppressor whose expression is reduced in NPC tissues and is absent in NPC metastases. RKIP expression altered the radiosensitivity of NPC cells through MEK and ERK phosphorylation changes of Raf-1/MEK/ERK signaling pathway. Down expression of RKIP was significantly correlated with advanced clinical stage, lymph node metastasis and radioresistance. Furthermore, survival curves showed that patients with RKIP down expression had a poor prognosis and induced relapse. Multivariate analysis confirmed that RKIP expression was an independent prognostic indicator. Ruan and colleagues suggested that RKIP may be a biomarker for the radiation sensitivity and prognosis of NPC. The results could help to provide new adjuvant therapies with radiotherapy for individual radioresistant NPC patients [121].

##### 1) Molecular targeted therapy in development: RKIP

RKIP as a biomarker for the radiation sensitivity of NPC could provide for the development of new molecular targeted therapy with radiotherapy for individual radioresistant NPC patients (Table 1).

#### Ras association domain family (RASSF)

Ras proteins are key signal transducers for various important pathways, such as PI3K, MAPK, Rho GTPases. Over the last few years, a group of proteins with Ras binding domain were identified as negative effectors of Ras. They were designated as RASSF. Wang et al investigated the mechanisms of RASSF1A as a tumor suppressor gene in NPC. They found that promoter methylation of RASSF1A could be detected in 71.05% (27/38) of NPC samples, but not in normal nasopharyngeal epithelia. RASSF1A expression in NPC primary tumors was lower than that in normal nasopharyngeal epithelial (P < 0.01) [122]. RASSF2 has been shown to bind directly with K-Ras in a GTP-dependent manner and its growth inhibition effect could be enhanced in the presence of activated Ras. RASSF2A is the only isoform of RASSF2 that contains CpG islands in its promoter and it has been reported to be inactivated by its promoter methylation in several human cancers. Zhang et al investigated the correlation of RASSF2A expression with its promoter methylation in NPC. Expression of RASSF2A was downregulated in 80% (4/5) of NPC cell lines. Decreased RASSF2A expression was also observed in NPC primary tumors compared with normal nasopharyngeal epithelia. RASSF2A-methylated cases showed a significantly lower level of RASSF2A expression than unmethylated cases. Patients with methylated RASSF2A presented a higher frequency of lymph node metastasis (P < 0.05) [123]. RASSF2A methylation can be considered as such an indicator of the propensity of lymph node metastasis, and would have a great potential in clinical application. In addition, methylation-mediated inactivation is potentially a reversible phenomenon. Turning this process around and up-regulating RASSF2A may probably prevent or reverse the malignant and metastatic phenotype, and therefore become a novel therapeutic target in NPC treatment.

##### 1) Molecular targeted therapy in development: demethylation of RASSF2A

Demethylation of RASSF2A and up-regulating RASSF2A may probably prevent or reverse the malignant and metastatic phenotype, and therefore become a novel therapeutic target in NPC treatment (Table 1).

### Receptor-mediated aberrations

#### Pigment epithelium-derived factor (PEDF)

PEDF is a potent and versatile endogenous inhibitor of angiogenesis [124]. A heterogenic xenografted human NPC nude mice model was established to investigate the effect of PEDF, and the combined effect of PEDF and radiotherapy on NPC. PEDF remarkably suppressed the growth of NPC by 43.52% and decreased the tumor microvessel density (MVD). PEDF decreased VEGF in NPC cell lines by downregulation of HIF-1α, a crucial transcriptional factor for VEGF expression. Xu and colleagues demonstrated that the combination of PEDF with radiotherapy enhances the efficacy of the antitumor effect on NPC by the coordinated inhibition on angiogenesis, which implies the potential role of PEDF as an adjuvant agent for NPC treatment [125].

#### VEGF-C

The contribution of the lymphatic system to tumor metastasis is being increasingly appreciated through studies of human cancers. As the biological behavior of NPC depends on its nodal status, patients with advanced nodal status show a higher tendency toward a poor outcome. Wakisaka et al reported that the lymphatic vessel counts (LVC) and VEGF-C expression were significantly higher in cases with advanced regional lymph node metastasis (N2,3) than those with no or limited lymph node involvement (N0,1) (P = 0.0380 and P = 0.0109, respectively) [126].

### Cell cycle aberrations

#### C-myc levels

C-myc is critical to the regulation of several important G1/S phase proteins; it sequesters inhibitory p27 from the CDK2/cyclin E complex, thus allowing for cell proliferation and progression through the G1 phase (Nesbit et al 1999). The study of Yu and colleagues showed that patients with c-Myc positive nasopharyngeal tumors had lower recurrence and death rates (38% (11/29) and 34% (10/29), respectively) than those with c-Myc negative tumors (73% (16/22) and 64% (14/22), respectively) [127]. The study of Hwang et al showed that decreased c-myc levels are found in 60% of NPC tumors and correlate with more aggressive NPC tumors with higher rates of lymph node metastasis [83]. This study also showed that poorly differentiated (types 2 and 3) NPC is more associated with low c-Myc levels, whereas well-differentiated (type 1) NPC has higher c-Myc levels.

The Myc protein is a transcription factor that regulates a variety of cellular processes including cell growth and proliferation, cell cycle progression, differentiation, apoptosis, and cell motility. Potential strategies that either inhibit the proliferation-promoting effect of Myc and/or activate its pro-apoptotic function are presently being explored [128].

##### 1) Molecular targeted therapy in development: inhibition of the effect of Myc

Potential strategies that either inhibit the effect of Myc and/or activate its pro-apoptotic function are presently being explored.

#### Galectin-3

Galectin-3 was shown to be involved in various biological events, including cell growth, adhesion, differentiation, angiogenesis, apoptosis, tumorigenesis, and metastasis. Acikalin and colleagues evaluated the expression of cyclin D1 and galectin-3 using immunohistochemical analysis in 45 patients diagnosed as undifferentiated NPC. Multivariate analysis showed that older age (> 50 vs. ≤ 50) (P = 0.028), distant metastasis at presentation (M1 vs. M0) (P = 0.001), and increased galectin-3 expression (> 5% vs. ≤ 5%) (P = 0.025) were independently correlated with poor OS. The Spearman’s correlation coefficient revealed a significant correlation between galectin-3 and cyclin D1 expression (r = 0.425; P = 0.004). These findings suggested that the immunohistochemical analysis of galectin-3 might be useful in predicting prognosis in NPC [129].

#### MicroRNA (miRNA) miR-218

MiRNAs represent a class of naturally occurring small non-coding RNA molecules. They regulate gene expression at the post-transcriptional level and control thereby cellular mechanisms including developmental transitions, organ morphology, apoptosis and cell proliferation. As might be expected from molecules with these roles, miRNAs are involved in cancer development, and deregulation of several miRNAs has been found in various cancer types [130]. Wong et al demonstrated that miRNAs of let-7 family suppress NPC cell proliferation through downregulating c-Myc expression [131]. Frequent downregulation of the microRNA miR-218 plays a critical role in NPC progression. Suppression of miR-218 was associated with epigenetic silencing of SLIT2 and SLIT3, ligands of ROBO receptors that have been previously implicated in tumor angiogenesis. Exogenous expression of miR-218 caused significant toxicity in NPC cells *in vitro* and delayed tumor growth *in vivo*. In clinical specimens of NPC (n = 71), Alajez and colleagues demonstrated that ROBO1 overexpression was significantly associated with worse overall (P = 0.04, HR = 2.4) and nodal relapse-free survival (P = 0.008, HR = 6.0). These findings defined an integrative tumor suppressor function for miR-218 in NPC and further suggested that restoring miR-218 expression in NPC might be useful for its clinical management [132].

##### 1) Molecular targeted therapy in development: miRNAs

MiRNAs of let-7 family suppress NPC cell proliferation (Table 1).

### Checkpoint with forkhead-associated and ring finger domains (CHFR)

CHFR is a mitotic checkpoint regulator that delays chromosome condensation in case of abnormal spindle formation [133]. Epigenetic inactivation of CHFR via aberrant hypermethylation of promoter region is frequently observed in many cancer cell lines and primary tumors [134]. In the study of Cheung and colleagues, hypermethylation of CHFR promoter CpG was detected in 100% NPC cell lines and xenografts (11 out of 11) and in 61.1% (22 out of 36) of NPC samples, but not in nonmalignant nasopharyngeal cell lines or tissues. The result indicates that downregulation of CHFR may play a role in the development of NPC. Undetectable or reduced expression of CHFR mRNA was also correlated with hypermethylation in all NPC cell lines as well as human NPC xenografts. Cheung and colleagues also found that eight NPC cell lines (100%) showed decreased or undetectable levels of CHFR mRNA which correlated to the aberrant hypermethylation of CpG islands in CHFR promoter region. More importantly, hypermethylation of CHFR promoter was detected in 61.1% (22 out of 36) of primary NPC tumors, implicating that inactivation of CHFR through methylation is common and will probably play an important role in the development of NPC [134]. Huang et al reported that the methylation frequency of CHFR in NPC primary tumors and their paired swabs were 65.5% and 63.8%, respectively [135]. Epigenetic inactivation of CHFR may be used as a diagnostic marker to monitor disease progression and recurrence. Downregulation of CHFR may serve as a molecular marker for predicting cellular sensitivity to certain microtubule disrupting anticancer drugs [134]. Moreover, aberrant DNA methylation has been recognized to be associated with the transcriptional inactivation of genes related to cancer drug resistance development. In order to identify the mechanism of DNA methylation involved in NPC taxol resistance, Zhang and colleague applied a genome-wide DNA methylation microarray assay to reveal methylation alteration in taxol-resistant NPC cell lines. Forty-eight differentially methylated genes were further identified in the three taxol-resistant cell lines. Six of them (CHFR, DLC1, ABCC5, PEG10, ERBB2, and GSTP1) were independently confirmed to contribute to taxol resistance by both methylation-specific PCR and quantitative real-time PCR. The authors conclude that DNA methylation is closely correlated with taxol drug resistance in NPC cells. Combined analysis of DNA methylation and gene expression may enable the discovery of new prognostic biomarkers of cancers and novel therapeutic targets [136].

### Centromere aberration

#### Centromere protein H (CENP-H)

CENP-H is one of the fundamental components of the human active kinetochore. The kinetochore is a large multiprotein complex that is assembled on centromeric DNA. It serves as the attachment site for spindle microtubules. Several kinetochore proteins have been identified in humans, including CENP-A, CENP-B, CENP-C, CENP-E, CENP-F, CENP-H, CENP-1, and INCENP. Kinetochore malfunction is a major cause of aneuploidy and is closely associated with carcinogenesis [137]. The overexpression of CENP-H has been shown to be closely associated with human cancers [138]. By immunohistochemical analysis, Liao et al found that 76 of 160 (47.5%) paraffin-embedded archival NPC biopsies showed high expression of CENP-H. Statistical analysis showed that there was a significant difference of CENP-H expression in patients categorized according to clinical stage (P = 0.024) and T classification (P = 0.027). Patients with higher CENP-H expression had shorter OS time, whereas patients with lower CENP-H expression had better survival. Multivariate analysis showed that CENP-H expression was an independent prognostic indicator for patient’s survival [137].

## Conclusion

The majority of newly diagnosed NPC patients have loco-regionally advanced disease [139]. A standard therapy for patients with loco-regionally advanced NPC is concurrent cisplatin chemotherapy followed by adjuvant chemotherapy. Although whether adjuvant chemotherapy is necessary has been debated, experts agree that cisplatin given concurrently with radiation improves OS [21]. The 5-year survival rate after treatment is approximately 70% [15, 140, 141]. Thirty to forty percent of patients will develop distant metastasis within 4 years [142], and once metastasis occurs, the prognosis is very poor.

In the era of new targeted molecules, two phase 2 trials of the addition of targeted agents to cisplatin and IMRT have been done. The first, done by Ma and colleagues in Hong Kong, used cetuximab to target EGFR, whereas the second, done by the Radiation Therapy Oncology Group (RTOG) used bevacizumab to exploit the angiogenesis pathway. Both trials used IMRT and although Ma and colleagues gave cisplatin once a week, the RTOG group used the standard regimen of cisplatin administered once every 3 weeks before adjuvant treatment with cisplatin and fluorouracil. In terms of concurrent cisplatin delivered, half the patients in Ma and colleagues’ trial received at least six cycles of cisplatin (30 mg/m^2^), whereas more than two-thirds of those in the RTOG study received three cycles of concurrent cisplatin (100 mg/m^2^).

Both agents seemed well tolerated, the main side-effect of cetuximab being acneiform rash, whereas bevacizumab was associated with only a slightly higher incidence of grade 1-2 bleeding. Severe (grade 3-4) acute mucositis was noted in 87% of patients in the Ma and colleagues’ study and just over three-quarters of those in the RTOG trial. Whether this difference is a factor of the frequency of cisplatin administration or the addition of the new targeted agent or a combination of both factors is not clear. Because control of distant metastasis should be the main objective of this strategy, it is encouraging to know that both trials resulted in 2-year DMFS of about 90% [143]. These promising results in early clinical trials should be validated in phase III clinical trials. Moreover, in order to further improve OS for patients with loco-regionally advanced NPC, the development of innovative strategies, including prognostic molecular markers and molecular targeted agents, is needed.

## References

[1] Hu C, Wei W, Chen X, Woodman CB, Yao Y, Nicholls JM, Joab I (2012). A global view of the oncogenic landscape in nasopharyngeal carcinoma: an integrated analysis at the genetic and expression levels. PLoS One.

[2] Chai SJ, Pua KC, Saleh A, Yap YY, Lim PV, Subramaniam SK, Lum CL (2012). Clinical significance of plasma Epstein-Barr Virus DNA loads in a large cohort of Malaysian patients with nasopharyngeal carcinoma. J Clin Virol.

[3] Ghosh SK, Perrine SP, Faller DV (2012). Advances in Virus-Directed Therapeutics against Epstein-Barr Virus-Associated Malignancies. Adv Virol.

[4] Thompson MP, Kurzrock R (2004). Epstein-Barr virus and cancer. Clin Cancer Res.

[5] Chen L, Hu CS, Chen XZ, Hu GQ, Cheng ZB, Sun Y, Li WX (2012). Concurrent chemoradiotherapy plus adjuvant chemotherapy versus concurrent chemoradiotherapy alone in patients with locoregionally advanced nasopharyngeal carcinoma: a phase 3 multicentre randomised controlled trial. Lancet Oncol.

[6] Li YH, Hu CF, Shao Q, Huang MY, Hou JH, Xie D, Zeng YX (2008). Elevated expressions of survivin and VEGF protein are strong independent predictors of survival in advanced nasopharyngeal carcinoma. J Transl Med.

[7] Chua DT, Nicholls JM, Sham JS, Au GK (2004). Prognostic value of epidermal growth factor receptor expression in patients with advanced stage nasopharyngeal carcinoma treated with induction chemotherapy and radiotherapy. Int J Radiat Oncol Biol Phys.

[8] Han L, Lin SJ, Pan JJ, Chen CB, Zhang Y, Zhang XC, Liao XY (2010). Prognostic factors of 305 nasopharyngeal carcinoma patients treated with intensity-modulated radiotherapy. Chin J Cancer.

[9] Leong JL, Loh KS, Putti TC, Goh BC, Tan LK (2004). Epidermal growth factor receptor in undifferentiated carcinoma of the nasopharynx. Laryngoscope.

[10] Ma BB, Poon TC, To KF, Zee B, Mo FK, Chan CM, Ho S (2003). Prognostic significance of tumor angiogenesis, Ki 67, p53 oncoprotein, epidermal growth factor receptor and HER2 receptor protein expression in undifferentiated nasopharyngeal carcinoma—a prospective study. Head Neck.

[11] Pan J, Kong L, Lin S, Chen G, Chen Q, Lu JJ (2008). The clinical significance of coexpression of cyclooxygenases-2, vascular endothelial growth factors, and epidermal growth factor receptor in nasopharyngeal carcinoma. Laryngoscope.

[12] Cao XJ, Hao JF, Yang XH, Xie P, Liu LP, Yao CP, Xu J (2012). Prognostic value of expression of EGFR and nm23 for locoregionally advanced nasopharyngeal carcinoma. Med Oncol.

[13] Yang Y, Xuan J, Yang Z, Han A, Xing L, Yue J, Hu M (2012). The expression of epidermal growth factor receptor and Ki67 in primary and relapse nasopharyngeal cancer: a micro-evidence for anti-EGFR targeted maintenance therapy. Med Oncol.

[14] Bonner JA, Harari PM, Giralt J, Azarnia N, Shin DM, Cohen RB, Jones CU (2006). Radiotherapy plus cetuximab for squamous-cell carcinoma of the head and neck. N Engl J Med.

[15] Chan AT, Leung SF, Ngan RK, Teo PM, Lau WH, Kwan WH, Hui EP (2005). Overall survival after concurrent cisplatin-radiotherapy compared with radiotherapy alone in locoregionally advanced nasopharyngeal carcinoma. J Natl Cancer Inst.

[16] Chan AT, Hsu MM, Goh BC, Hui EP, Liu TW, Millward MJ, Hong RL (2005). Multicenter, phase II study of cetuximab in combination with carboplatin in patients with recurrent or metastatic nasopharyngeal carcinoma. J Clin Oncol.

[17] Chua DT, Wei WI, Wong MP, Sham JS, Nicholls J, Au GK (2008). Phase II study of gefitinib for the treatment of recurrent and metastatic nasopharyngeal carcinoma. Head Neck.

[18] Ma B, Hui EP, King A, To KF, Mo F, Leung SF, Kam M (2008). A phase II study of patients with metastatic or locoregionally recurrent nasopharyngeal carcinoma and evaluation of plasma Epstein-Barr virus DNA as a biomarker of efficacy. Cancer Chemother Pharmacol.

[19] Hsieh WS, Soo R, Peh BK, Loh T, Dong D, Soh D, Wong LS (2009). Pharmacodynamic effects of seliciclib, an orally administered cell cycle modulator, in undifferentiated nasopharyngeal cancer. Clin Cancer Res.

[20] Ma BB, Kam MK, Leung SF, Hui EP, King AD, Chan SL, Mo F (2012). A phase II study of concurrent cetuximab-cisplatin and intensity-modulated radiotherapy in locoregionally advanced nasopharyngeal carcinoma. Ann Oncol.

[21] Lee NY, Zhang Q, Pfister DG, Kim J, Garden AS, Mechalakos J, Hu K (2012). Addition of bevacizumab to standard chemoradiation for locoregionally advanced nasopharyngeal carcinoma (RTOG 0615): a phase 2 multi-institutional trial. Lancet Oncol.

[22] Lv X, Xiang YQ, Cao SM, Qian CN, Li NW, Guo L, Mai HQ (2011). Prospective validation of the prognostic value of elevated serum vascular endothelial growth factor in patients with nasopharyngeal carcinoma: more distant metastases and shorter overall survival after treatment. Head Neck.

[23] Wakisaka N, Wen QH, Yoshizaki T, Nishimura T, Furukawa M, Kawahara E, Nakanishi I (1999). Association of vascular endothelial growth factor expression with angiogenesis and lymph node metastasis in nasopharyngeal carcinoma. Laryngoscope.

[24] Krishna SM, James S, Balaram P (2006). Expression of VEGF as prognosticator in primary nasopharyngeal cancer and its relation to EBV status. Virus Res.

[25] Chang KP, Chang YT, Wu CC, Liu YL, Chen MC, Tsang NM, Hsu CL (2011). Multiplexed immunobead-based profiling of cytokine markers for detection of nasopharyngeal carcinoma and prognosis of patient survival. Head Neck.

[26] Lo YM, Chan AT, Chan LY, Leung SF, Lam CW, Huang DP, Johnson PJ (2000). Molecular prognostication of nasopharyngeal carcinoma by quantitative analysis of circulating Epstein-Barr virus DNA. Cancer Res.

[27] Leung SF, Zee B, Ma BB, Hui EP, Mo F, Lai M, Chan KC (2006). Plasma Epstein-Barr viral deoxyribonucleic acid quantitation complements tumor-node-metastasis staging prognostication in nasopharyngeal carcinoma. J Clin Oncol.

[28] Lin JC, Wang WY, Chen KY, Wei YH, Liang WM, Jan JS, Jiang RS (2004). Quantification of plasma Epstein-Barr virus DNA in patients with advanced nasopharyngeal carcinoma. N Engl J Med.

[29] Ferrari D, Codeca C, Bertuzzi C, Broggio F, Crepaldi F, Luciani A, Floriani I (2012). Role of plasma EBV DNA levels in predicting recurrence of nasopharyngeal carcinoma in a Western population. BMC Cancer.

[30] Li SW, Wang H, Xiang YQ, Zhang HB, Lv X, Xia WX, Zeng MS (2013). Prospective study of prognostic value of Raf kinase inhibitory protein and pretreatment plasma Epstein-Barr virus DNA for distant metastasis in locoregionally advanced nasopharyngeal carcinoma. Head Neck.

[31] Lin YC, You L, Xu Z, He B, Mikami I, Thung E, Chou J (2006). Wnt signaling activation and WIF-1 silencing in nasopharyngeal cancer cell lines. Biochem Biophys Res Commun.

[32] Li LL, Shu XS, Wang ZH, Cao Y, Tao Q (2011). Epigenetic disruption of cell signaling in nasopharyngeal carcinoma. Chin J Cancer.

[33] Shu XS, Geng H, Li L, Ying J, Ma C, Wang Y, Poon FF (2011). The epigenetic modifier PRDM5 functions as a tumor suppressor through modulating WNT/beta-catenin signaling and is frequently silenced in multiple tumors. PLoS One.

[34] Fendri A, Khabir A, Hadri-Guiga B, Sellami-Boudawara T, Daoud J, Frikha M, Ghorbel A (2010). Epigenetic alteration of the Wnt inhibitory factor-1 promoter is common and occurs in advanced stage of Tunisian nasopharyngeal carcinoma. Cancer Invest.

[35] Hong B, Lui VW, Hui EP, Lu Y, Leung HS, Wong EY, Cheng SH (2010). Reverse phase protein array identifies novel anti-invasion mechanisms of YC-1. Biochem Pharmacol.

[36] Hernandez-Aya LF, Gonzalez-Angulo AM (2011). Targeting the phosphatidylinositol 3-kinase signaling pathway in breast cancer. Oncologist.

[37] Or YY, Hui AB, To KF, Lam CN, Lo KW (2006). PIK3CA mutations in nasopharyngeal carcinoma. Int J Cancer.

[38] Fendri A, Khabir A, Mnejja W, Sellami-Boudawara T, Daoud J, Frikha M, Ghorbel A (2009). PIK3CA amplification is predictive of poor prognosis in Tunisian patients with nasopharyngeal carcinoma. Cancer Sci.

[39] Jiang H, Fan D, Zhou G, Li X, Deng H (2010). Phosphatidylinositol 3-kinase inhibitor(LY294002) induces apoptosis of human nasopharyngeal carcinoma in vitro and in vivo. J Exp Clin Cancer Res.

[40] Yang L, Lu Z, Ma X, Cao Y, Sun LQ (2010). A therapeutic approach to nasopharyngeal carcinomas by DNAzymes targeting EBV LMP-1 gene. Molecules.

[41] Wang D, Liebowitz D, Kieff E (1985). An EBV membrane protein expressed in immortalized lymphocytes transforms established rodent cells. Cell.

[42] Mainou BA, Raab-Traub N (2006). LMP1 strain variants: biological and molecular properties. J Virol.

[43] Ozyar E, Ayhan A, Korcum AF, Atahan IL (2004). Prognostic role of Ebstein-Barr virus latent membrane protein-1 and interleukin-10 expression in patients with nasopharyngeal carcinoma. Cancer Invest.

[44] Liu LT, Peng JP, Chang HC, Hung WC (2003). RECK is a target of Epstein-Barr virus latent membrane protein 1. Oncogene.

[45] Chew MM, Gan SY, Khoo AS, Tan EL (2010). Interleukins, laminin and Epstein - Barr virus latent membrane protein 1 (EBV LMP1) promote metastatic phenotype in nasopharyngeal carcinoma. BMC Cancer.

[46] Chou J, Lin YC, Kim J, You L, Xu Z, He B, Jablons DM (2008). Nasopharyngeal carcinoma—review of the molecular mechanisms of tumorigenesis. Head Neck.

[47] Lan YY, Hsiao JR, Chang KC, Chang JS, Chen CW, Lai HC, Wu SY (2012). Epstein-Barr virus latent membrane protein 2A promotes invasion of nasopharyngeal carcinoma cells through ERK/Fra-1-mediated induction of matrix metalloproteinase 9. J Virol.

[48] Chen J, Hu CF, Hou JH, Shao Q, Yan LX, Zhu XF, Zeng YX (2010). Epstein-Barr virus encoded latent membrane protein 1 regulates mTOR signaling pathway genes which predict poor prognosis of nasopharyngeal carcinoma. J Transl Med.

[49] Zhao Y, Wang Y, Zeng S, Hu X (2012). LMP1 expression is positively associated with metastasis of nasopharyngeal carcinoma: evidence from a meta-analysis. J Clin Pathol.

[50] Rankin EB, Giaccia AJ (2008). The role of hypoxia-inducible factors in tumorigenesis. Cell Death Differ.

[51] Semenza G (2002). Signal transduction to hypoxia-inducible factor 1. Biochem Pharmacol.

[52] Ma BB, Hui EP, Chan AT (2008). Systemic approach to improving treatment outcome in nasopharyngeal carcinoma: current and future directions. Cancer Sci.

[53] Hui EP, Chan AT, Pezzella F, Turley H, To KF, Poon TC, Zee B (2002). Coexpression of hypoxia-inducible factors 1alpha and 2alpha, carbonic anhydrase IX, and vascular endothelial growth factor in nasopharyngeal carcinoma and relationship to survival. Clin Cancer Res.

[54] Chan CM, Ma BB, Hui EP, Wong SC, Mo FK, Leung SF, Kam MK (2007). Cyclooxygenase-2 expression in advanced nasopharyngeal carcinoma—a prognostic evaluation and correlation with hypoxia inducible factor 1alpha and vascular endothelial growth factor. Oral Oncol.

[55] Xueguan L, Xiaoshen W, Yongsheng Z, Chaosu H, Chunying S, Yan F (2008). Hypoxia inducible factor-1 alpha and vascular endothelial growth factor expression are associated with a poor prognosis in patients with nasopharyngeal carcinoma receiving radiotherapy with carbogen and nicotinamide. Clin Oncol (R Coll Radiol).

[56] Wan XB, Fan XJ, Chen MY, Xiang J, Huang PY, Guo L, Wu XY (2010). Elevated Beclin 1 expression is correlated with HIF-1alpha in predicting poor prognosis of nasopharyngeal carcinoma. Autophagy.

[57] Shou Z, Lin L, Liang J, Li JL, Chen HY (2012). Expression and prognosis of FOXO3a and HIF-1alpha in nasopharyngeal carcinoma. J Cancer Res Clin Oncol.

[58] Baker LC, Boult JK, Walker-Samuel S, Chung YL, Jamin Y, Ashcroft M, Robinson SP (2012). The HIF-pathway inhibitor NSC-134754 induces metabolic changes and anti-tumour activity while maintaining vascular function. Br J Cancer.

[59] Shen Y, Wu Y, Chen M, Shen W, Huang S, Zhang L, Zou X (2012). Effects of pantoprazole as a HIF-1alpha inhibitor on human gastric adenocarcinoma sgc-7901 cells. Neoplasma.

[60] Kim YJ, Go H, Wu HG, Jeon YK, Park SW, Lee SH (2011). Immunohistochemical study identifying prognostic biomolecular markers in nasopharyngeal carcinoma treated by radiotherapy. Head Neck.

[61] Razak AR, Siu LL, Liu FF, Ito E, O'Sullivan B, Chan K (2010). Nasopharyngeal carcinoma: the next challenges. Eur J Cancer.

[62] Jo M, Stolz DB, Esplen JE, Dorko K, Michalopoulos GK, Strom SC (2000). Cross-talk between epidermal growth factor receptor and c-Met signal pathways in transformed cells. J Biol Chem.

[63] Eder JP, Vande Woude GF, Boerner SA, LoRusso PM (2009). Novel therapeutic inhibitors of the c-Met signaling pathway in cancer. Clin Cancer Res.

[64] Yu H, Li X, Sun S, Gao X, Zhou D (2012). c-Met inhibitor SU11274 enhances the response of the prostate cancer cell line DU145 to ionizing radiation. Biochem Biophys Res Commun.

[65] Wiehr S, von Ahsen O, Rose L, Mueller A, Mannheim JG, Honndorf V, Kukuk D (2013). Preclinical evaluation of a novel c-Met inhibitor in a gastric cancer xenograft model using small animal PET. Mol Imaging Biol.

[66] Lee NV, Lira ME, Pavlicek A, Ye J, Buckman D, Bagrodia S, Srinivasa SP (2012). A novel SND1-BRAF fusion confers resistance to c-Met inhibitor PF-04217903 in GTL16 cells through [corrected] MAPK activation. PLoS One.

[67] Horikawa T, Sheen TS, Takeshita H, Sato H, Furukawa M, Yoshizaki T (2001). Induction of c-Met proto-oncogene by Epstein-Barr virus latent membrane protein-1 and the correlation with cervical lymph node metastasis of nasopharyngeal carcinoma. Am J Pathol.

[68] Qian CN, Guo X, Cao B, Kort EJ, Lee CC, Chen J, Wang LM (2002). Met protein expression level correlates with survival in patients with late-stage nasopharyngeal carcinoma. Cancer Res.

[69] Nobori T, Miura K, Wu DJ, Lois A, Takabayashi K, Carson DA (1994). Deletions of the cyclin-dependent kinase-4 inhibitor gene in multiple human cancers. Nature.

[70] Stone S, Jiang P, Dayananth P, Tavtigian SV, Katcher H, Parry D, Peters G (1995). Complex structure and regulation of the P16 (MTS1) locus. Cancer Res.

[71] Herman JG, Merlo A, Mao L, Lapidus RG, Issa JP, Davidson NE, Sidransky D (1995). Inactivation of the CDKN2/p16/MTS1 gene is frequently associated with aberrant DNA methylation in all common human cancers. Cancer Res.

[72] Merlo A, Herman JG, Mao L, Lee DJ, Gabrielson E, Burger PC, Baylin SB (1995). 5' CpG island methylation is associated with transcriptional silencing of the tumour suppressor p16/CDKN2/MTS1 in human cancers. Nat Med.

[73] Kwong J, Lo KW, To KF, Teo PM, Johnson PJ, Huang DP (2002). Promoter hypermethylation of multiple genes in nasopharyngeal carcinoma. Clin Cancer Res.

[74] Song X, Tao YG, Deng XY, Jin X, Tan YN, Tang M, Wu Q (2004). Heterodimer formation between c-Jun and Jun B proteins mediated by Epstein-Barr virus encoded latent membrane protein 1. Cell Signal.

[75] Wang L, Yao L, Zhang S, Liang C, Cheng N (1999). [Relationship between expression of P16 protein and prognosis in carcinoma of nasopharynx]. Hua Xi Yi Ke Da Xue Xue Bao.

[76] Makitie AA, MacMillan C, Ho J, Shi W, Lee A, O'Sullivan B, Payne D (2003). Loss of p16 expression has prognostic significance in human nasopharyngeal carcinoma. Clin Cancer Res.

[77] Xiang YN, Zhang WY (2005). [The clinical significance of p16 protein non-expression and p16 gene inactivation by deletions and hypermethylation in nasopharyngeal carcinoma]. Zhonghua Bing Li Xue Za Zhi.

[78] Kudo Y, Kitajima S, Ogawa I, Miyauchi M, Takata T (2005). Down-regulation of Cdk inhibitor p27 in oral squamous cell carcinoma. Oral Oncol.

[79] Tomoda K, Kubota Y, Kato J (1999). Degradation of the cyclin-dependent-kinase inhibitor p27Kip1 is instigated by Jab1. Nature.

[80] Dahinden C, Ingold B, Wild P, Boysen G, Luu VD, Montani M, Kristiansen G (2010). Mining tissue microarray data to uncover combinations of biomarker expression patterns that improve intermediate staging and grading of clear cell renal cell cancer. Clin Cancer Res.

[81] Pan Y, Zhang Q, Tian L, Wang X, Fan X, Zhang H, Claret FX (2012). Jab1/CSN5 negatively regulates p27 and plays a role in the pathogenesis of nasopharyngeal carcinoma. Cancer Res.

[82] Baba Y, Tsukuda M, Mochimatsu I, Furukawa S, Kagata H, Satake K, Koshika S (2001). Reduced expression of p16 and p27 proteins in nasopharyngeal carcinoma. Cancer Detect Prev.

[83] Hwang CF, Su CY, Huang SC, Huang CC, Fang FM, Lui CC, Chang HW (2003). Low expression levels of p27 correlate with loco-regional recurrence in nasopharyngeal carcinoma. Cancer Lett.

[84] Chow NH, Liu HS, Chan SH (2000). The role of nm23-H1 in the progression of transitional cell bladder cancer. Clin Cancer Res.

[85] Guo X, Min HQ, Zeng MS, Qian CN, Huang XM, Shao JY, Hou JH (1998). nm23-H1 expression in nasopharyngeal carcinoma: correlation with clinical outcome. Int J Cancer.

[86] Huang GW, Mo WN, Kuang GQ, Nong HT, Wei MY, Sunagawa M, Kosugi T (2001). Expression of p16, nm23-H1, E-cadherin, and CD44 gene products and their significance in nasopharyngeal carcinoma. Laryngoscope.

[87] Shih LC, Tsai CW, Tsai MH, Tsou YA, Chang WS, Li FJ, Lee MH (2012). Association of cyclin D1 genotypes with nasopharyngeal carcinoma risk. Anticancer Res.

[88] Huang XM, Dai CB, Mou ZL, Wang LJ, Wen WP, Lin SG, Xu G (2009). Overproduction of cyclin D1 is dependent on activated mTORC1 signal in nasopharyngeal carcinoma: implication for therapy. Cancer Lett.

[89] Shi Y, Tao Y, Jiang Y, Xu Y, Yan B, Chen X, Xiao L (2012). Nuclear epidermal growth factor receptor interacts with transcriptional intermediary factor 2 to activate cyclin D1 gene expression triggered by the oncoprotein latent membrane protein 1. Carcinogenesis.

[90] Lai JP, Tong CL, Hong C, Xiao JY, Tao ZD, Zhang Z, Tong WM (2002). Association between high initial tissue levels of cyclin d1 and recurrence of nasopharyngeal carcinoma. Laryngoscope.

[91] Galimberti F, Thompson SL, Liu X, Li H, Memoli V, Green SR, DiRenzo J (2010). Targeting the cyclin E-Cdk-2 complex represses lung cancer growth by triggering anaphase catastrophe. Clin Cancer Res.

[92] Ko MT, Su CY, Huang SC, Chen CH, Hwang CF (2009). Overexpression of cyclin E messenger ribonucleic acid in nasopharyngeal carcinoma correlates with poor prognosis. J Laryngol Otol.

[93] Vleminckx K, Vakaet L, Mareel M, Fiers W, van Roy F (1991). Genetic manipulation of E-cadherin expression by epithelial tumor cells reveals an invasion suppressor role. Cell.

[94] Li Z, Ren Y, Lin SX, Liang YJ, Liang HZ (2004). Association of E-cadherin and beta-catenin with metastasis in nasopharyngeal carcinoma. Chin Med J (Engl).

[95] Ran Y, Wu S, You Y (2011). Demethylation of E-cadherin gene in nasopharyngeal carcinoma could serve as a potential therapeutic strategy. J Biochem.

[96] Galera-Ruiz H, Rios MJ, Gonzalez-Campora R, de Miguel M, Carmona MI, Moreno AM, Galera-Davidson H (2011). The cadherin-catenin complex in nasopharyngeal carcinoma. Eur Arch Otorhinolaryngol.

[97] Vihinen P, Ala-aho R, Kahari VM (2005). Matrix metalloproteinases as therapeutic targets in cancer. Curr Cancer Drug Targets.

[98] Grimm M, Lazariotou M, Kircher S, Stuermer L, Reiber C, Hofelmayr A, Gattenlohner S (2010). MMP-1 is a (pre-)invasive factor in Barrett-associated esophageal adenocarcinomas and is associated with positive lymph node status. J Transl Med.

[99] Horikawa T, Yoshizaki T, Sheen TS, Lee SY, Furukawa M (2000). Association of latent membrane protein 1 and matrix metalloproteinase 9 with metastasis in nasopharyngeal carcinoma. Cancer.

[100] Wong TS, Kwong DL, Sham JS, Wei WI, Kwong YL, Yuen AP (2004). Clinicopathologic significance of plasma matrix metalloproteinase-2 and -9 levels in patients with undifferentiated nasopharyngeal carcinoma. Eur J Surg Oncol.

[101] Nasr HB, Mestiri S, Chahed K, Bouaouina N, Gabbouj S, Jalbout M, Chouchane L (2007). Matrix metalloproteinase-1 (-1607) 1G/2G and -9 (-1562) C/T promoter polymorphisms: susceptibility and prognostic implications in nasopharyngeal carcinomas. Clin Chim Acta.

[102] Liu Z, Li L, Yang Z, Luo W, Li X, Yang H, Yao K (2010). Increased expression of MMP9 is correlated with poor prognosis of nasopharyngeal carcinoma. BMC Cancer.

[103] He JR, Qin H, Ren ZF, Cui C, Zhang Y, Ranatunga D, Zeng YX (2011). MMP-9 expression in peripheral blood mononuclear cells and the association with clinicopathological features and prognosis of nasopharyngeal carcinoma. Clin Chem Lab Med.

[104] Gialeli C, Theocharis AD, Karamanos NK (2011). Roles of matrix metalloproteinases in cancer progression and their pharmacological targeting. FEBS J.

[105] Jin Y, An X, Cai YC, Cao Y, Cai XY, Xia Q, Tan YT (2011). Zoledronic acid combined with chemotherapy bring survival benefits to patients with bone metastases from nasopharyngeal carcinoma. J Cancer Res Clin Oncol.

[106] Wen QL, Meng MB, Yang B, Tu LL, Jia L, Zhou L, Xu Y (2009). Endostar, a recombined humanized endostatin, enhances the radioresponse for human nasopharyngeal carcinoma and human lung adenocarcinoma xenografts in mice. Cancer Sci.

[107] Peng F, Xu Z, Wang J, Chen Y, Li Q, Zuo Y, Chen J (2012). Recombinant human endostatin normalizes tumor vasculature and enhances radiation response in xenografted human nasopharyngeal carcinoma models. PLoS One.

[108] Ma F, Zhang H, Zhai Y, Huang W, Zhao C, Ou S, Zhou H (2011). Functional polymorphism -31C/G in the promoter of BIRC5 gene and risk of nasopharyngeal carcinoma among chinese. PLoS One.

[109] Ai MD, Li LL, Zhao XR, Wu Y, Gong JP, Cao Y (2005). Regulation of survivin and CDK4 by Epstein-Barr virus encoded latent membrane protein 1 in nasopharyngeal carcinoma cell lines. Cell Res.

[110] Faqing T, Zhi H, Liqun Y, Min T, Huanhua G, Xiyun D, Ya C (2005). Epstein-Barr virus LMP1 initiates cell proliferation and apoptosis inhibition via regulating expression of Survivin in nasopharyngeal carcinoma. Exp Oncol.

[111] Xiang Y, Yao H, Wang S, Hong M, He J, Cao S, Min H (2006). Prognostic value of Survivin and Livin in nasopharyngeal carcinoma. Laryngoscope.

[112] Hwang CF, Chien CY, Huang SC, Yin YF, Huang CC, Fang FM, Tsai HT (2010). Fibulin-3 is associated with tumour progression and a poor prognosis in nasopharyngeal carcinomas and inhibits cell migration and invasion via suppressed AKT activity. J Pathol.

[113] Li J, Yen C, Liaw D, Podsypanina K, Bose S, Wang SI, Puc J (1997). PTEN, a putative protein tyrosine phosphatase gene mutated in human brain, breast, and prostate cancer. Science.

[114] Maehama T, Dixon JE (1998). The tumor suppressor, PTEN/MMAC1, dephosphorylates the lipid second messenger, phosphatidylinositol 3,4,5-trisphosphate. J Biol Chem.

[115] Wong AM, Kong KL, Tsang JW, Kwong DL, Guan XY (2012). Profiling of Epstein-Barr virus-encoded microRNAs in nasopharyngeal carcinoma reveals potential biomarkers and oncomirs. Cancer.

[116] Xu X, Yang H, Huo X (2004). [Expression and significance of PTEN in nasopharyngeal carcinoma]. Lin Chuang Er Bi Yan Hou Ke Za Zhi.

[117] Song LB, Li J, Liao WT, Feng Y, Yu CP, Hu LJ, Kong QL (2009). The polycomb group protein Bmi-1 represses the tumor suppressor PTEN and induces epithelial-mesenchymal transition in human nasopharyngeal epithelial cells. J Clin Invest.

[118] Song LB, Zeng MS, Liao WT, Zhang L, Mo HY, Liu WL, Shao JY (2006). Bmi-1 is a novel molecular marker of nasopharyngeal carcinoma progression and immortalizes primary human nasopharyngeal epithelial cells. Cancer Res.

[119] Lo AK, Liu Y, Wang XH, Huang DP, Yuen PW, Wong YC, Tsao GS (2003). Alterations of biologic properties and gene expression in nasopharyngeal epithelial cells by the Epstein-Barr virus-encoded latent membrane protein 1. Lab Invest.

[120] Wang SS, Guan ZZ, Xiang YQ, Wang B, Lin TY, Jiang WQ, Zhang L (2006). [Significance of EGFR and p-ERK expression in nasopharyngeal carcinoma]. Zhonghua Zhong Liu Za Zhi.

[121] Ruan L, Wang GL, Yi H, Chen Y, Tang CE, Zhang PF, Li MY (2010). Raf kinase inhibitor protein correlates with sensitivity of nasopharyngeal carcinoma to radiotherapy. J Cell Biochem.

[122] Wang T, Liu H, Chen Y, Liu W, Yu J, Wu G (2009). Methylation associated inactivation of RASSF1A and its synergistic effect with activated K-Ras in nasopharyngeal carcinoma. J Exp Clin Cancer Res.

[123] Zhang Z, Sun D, Van do N, Tang A, Hu L, Huang G (2007). Inactivation of RASSF2A by promoter methylation correlates with lymph node metastasis in nasopharyngeal carcinoma. Int J Cancer.

[124] Yang J, Chen S, Huang X, Han J, Wang Q, Shi D, Cheng R (2010). Growth suppression of cervical carcinoma by pigment epithelium-derived factor via anti-angiogenesis. Cancer Biol Ther.

[125] Xu Z, Fang S, Zuo Y, Zhang Y, Cheng R, Wang Q, Yang Z (2011). Combination of pigment epithelium-derived factor with radiotherapy enhances the antitumor effects on nasopharyngeal carcinoma by downregulating vascular endothelial growth factor expression and angiogenesis. Cancer Sci.

[126] Wakisaka N, Hirota K, Kondo S, Sawada-Kitamura S, Endo K, Murono S, Yoshizaki T (2012). Induction of lymphangiogenesis through vascular endothelial growth factor-C/vascular endothelial growth factor receptor 3 axis and its correlation with lymph node metastasis in nasopharyngeal carcinoma. Oral Oncol.

[127] Yu Y, Dong W, Li X, Yu E, Zhou X, Li S (2003). Significance of c-Myc and Bcl-2 protein expression in nasopharyngeal carcinoma. Arch Otolaryngol Head Neck Surg.

[128] Grotzer MA, Castelletti D, Fiaschetti G, Shalaby T, Arcaro A (2009). Targeting Myc in pediatric malignancies of the central and peripheral nervous system. Curr Cancer Drug Targets.

[129] Acikalin MF, Etiz D, Gurbuz MK, Ozudogru E, Canaz F, Colak E (2012). Prognostic significance of galectin-3 and cyclin D1 expression in undifferentiated nasopharyngeal carcinoma. Med Oncol.

[130] Hummel R, Hussey DJ, Haier J (2010). MicroRNAs: predictors and modifiers of chemo- and radiotherapy in different tumour types. Eur J Cancer.

[131] Wong TS, Man OY, Tsang CM, Tsao SW, Tsang RK, Chan JY, Ho WK (2011). MicroRNA let-7 suppresses nasopharyngeal carcinoma cells proliferation through downregulating c-Myc expression. J Cancer Res Clin Oncol.

[132] Alajez NM, Lenarduzzi M, Ito E, Hui AB, Shi W, Bruce J, Yue S (2011). MiR-218 suppresses nasopharyngeal cancer progression through downregulation of survivin and the SLIT2-ROBO1 pathway. Cancer Res.

[133] Scolnick DM, Halazonetis TD (2000). Chfr defines a mitotic stress checkpoint that delays entry into metaphase. Nature.

[134] Cheung HW, Ching YP, Nicholls JM, Ling MT, Wong YC, Hui N, Cheung A (2005). Epigenetic inactivation of CHFR in nasopharyngeal carcinoma through promoter methylation. Mol Carcinog.

[135] Huang T, Du C, Yu N, Xiao X, Zhou X, Wang S, Huang G (2011). [Aberrant promoter hypermethylation of CHFR in nasopharyngeal carcinoma]. Lin Chung Er Bi Yan Hou Tou Jing Wai Ke Za Zhi.

[136] Zhang X, Li W, Li H, Ma Y, He G, Tan G (2012). Genomic methylation profiling combined with gene expression microarray reveals the aberrant methylation mechanism involved in nasopharyngeal carcinoma taxol resistance. Anticancer Drugs.

[137] Liao WT, Song LB, Zhang HZ, Zhang X, Zhang L, Liu WL, Feng Y Centromere protein H is a novel prognostic marker for nasopharyngeal carcinoma progression and overall patient survival. Clin Cancer Res. 2007;13(2 Pt.

[138] Zhao WF, Wang HB, Xie B, Hu LJ, Xu LH, Kuang BH, Li MZ (2012). Sp1 and Sp3 are involved in the full transcriptional activity of centromere protein H in human nasopharyngeal carcinoma cells. FEBS J.

[139] Mao YP, Xie FY, Liu LZ, Sun Y, Li L, Tang LL, Liao XB (2009). Re-evaluation of 6th edition of AJCC staging system for nasopharyngeal carcinoma and proposed improvement based on magnetic resonance imaging. Int J Radiat Oncol Biol Phys.

[140] Lee AW, Yau TK, Wong DH, Chan EW, Yeung RM, Ng WT, Tong M (2005). Treatment of stage IV(A-B) nasopharyngeal carcinoma by induction-concurrent chemoradiotherapy and accelerated fractionation. Int J Radiat Oncol Biol Phys.

[141] Lee AW, Tung SY, Ngan RK, Chappell R, Chua DT, Lu TX, Siu L (2011). Factors contributing to the efficacy of concurrent-adjuvant chemotherapy for locoregionally advanced nasopharyngeal carcinoma: combined analyses of NPC-9901 and NPC-9902 Trials. Eur J Cancer.

[142] Le QT, Tate D, Koong A, Gibbs IC, Chang SD, Adler JR, Pinto HA (2003). Improved local control with stereotactic radiosurgical boost in patients with nasopharyngeal carcinoma. Int J Radiat Oncol Biol Phys.

[143] Wee J (2012). Nasopharyngeal cancer: a promising future. Lancet Oncol.

